# *CBR3* V244M is associated with LVEF reduction in breast cancer patients treated with doxorubicin

**DOI:** 10.1186/s40959-021-00103-0

**Published:** 2021-05-11

**Authors:** Jennifer K. Lang, Badri Karthikeyan, Adolfo Quiñones-Lombraña, Rachael Hageman Blair, Amy P. Early, Ellis G. Levine, Umesh C. Sharma, Javier G. Blanco, Tracey O’Connor

**Affiliations:** 1 Department of Medicine, Division of Cardiology, Jacobs School of Medicine and Biomedical Sciences, Buffalo, NY 14203 USA; 2 Veterans Affairs Western New York Healthcare System, Buffalo, NY USA; 3 Department of Pharmaceutical Sciences, School of Pharmacy and Pharmaceutical Sciences, University at Buffalo, Buffalo, NY 14214 USA; 4 Department of Biostatistics, School of Public Health and Health Professions, University at Buffalo, Buffalo, NY 14214 USA; 5 Department of Medicine, Roswell Park Comprehensive Cancer Center, Buffalo, NY 14203 USA

**Keywords:** Anthracycline, Breast Cancer, Carbonyl Reductase 3, Cardiotoxicity, Cardiovascular disease, Chemotherapy, Doxorubicin, Survivorship

## Abstract

**Background:**

The *CBR3* V244M single nucleotide polymorphism has been linked to the risk of anthracycline-related cardiomyopathy in survivors of childhood cancer. There have been limited prospective studies examining the impact of *CBR3* V244M on the risk for anthracycline-related cardiotoxicity in adult cohorts.

**Objectives:**

This study evaluated the presence of associations between *CBR3* V244M genotype status and changes in echocardiographic parameters in breast cancer patients undergoing doxorubicin treatment.

**Methods:**

We recruited 155 patients with breast cancer receiving treatment with doxorubicin (DOX) at Roswell Park Comprehensive Care Center (Buffalo, NY) to a prospective single arm observational pharmacogenetic study. Patients were genotyped for the *CBR3* V244M variant. 92 patients received an echocardiogram at baseline (t_0 month_) and at 6 months (t_6 months_) of follow up after DOX treatment. Apical two-chamber and four-chamber echocardiographic images were used to calculate volumes and left ventricular ejection fraction (LVEF) using Simpson’s biplane rule by investigators blinded to all patient data. Volumetric indices were evaluated by normalizing the cardiac volumes to the body surface area (BSA).

**Results:**

Breast cancer patients with *CBR3* GG and AG genotypes both experienced a statistically significant reduction in LVEF at 6 months following initiation of DOX treatment for breast cancer compared with their pre-DOX baseline study. Patients homozygous for the *CBR3* V244M G allele (CBR3 V244) exhibited a further statistically significant decrease in LVEF at 6 months following DOX therapy in comparison with patients with heterozygous AG genotype. We found no differences in age, pre-existing cardiac diseases associated with myocardial injury, cumulative DOX dose, or concurrent use of cardioprotective medication between *CBR3* genotype groups.

**Conclusions:**

*CBR3* V244M genotype status is associated with changes in echocardiographic parameters suggestive of early anthracycline-related cardiomyopathy in subjects undergoing chemotherapy for breast cancer.

**Supplementary Information:**

The online version contains supplementary material available at 10.1186/s40959-021-00103-0.

## Introduction

Progress made in early cancer diagnosis and therapy has translated into increased longevity for patients with breast cancer. As survival has increased, the potential cardiotoxicity of cancer chemotherapy regimens has become an important issue for survivorship. Doxorubicin (DOX)-induced cardiotoxicity has been demonstrated at a cumulative dose of ≤300 mg/m^2^, with histopathological changes seen in endomyocardial biopsy tissue from patients receiving as little as 240 mg/m^2^ of DOX [1]. In cardiomyocytes, specific cytosolic carbonyl reductases (CBRs) and aldo-keto reductases (AKRs) convert the parent anthracycline to C-13 alcohol metabolites (e.g., doxorubicinol, DOXOL). These metabolites accumulate in long term reservoirs and play a key role in the pathogenesis of chemotherapy-induced cardiomyopathy. Anthracycline-induced cardiotoxicity is mainly mediated by increased formation of reactive oxygen species (ROS), perturbations in iron handling, and inhibition of topoisomerase 2β in cardiomyocytes leading to cell injury and death [2].

Individual risk stratification and early detection of chemotherapy-induced cardiotoxicity are crucial to prevent irreversible cardiac dysfunction. Our prior work with the Children’s Oncology Group identified a single nucleotide polymorphic variant in the *CBR3* gene (rs1056892, *CBR3* V244M) associated with the risk of anthracycline-related cardiomyopathy in survivors of childhood cancers [3]. The homozygous *CBR3* G genotype (CBR3 V244) was found to correlate with a 3.3-fold increased risk of cardiomyopathy following exposure to anthracyclines (1–250 mg/m^2^) compared with *CBR3* GA/AA genotypes [3]. Kinetic studies with isoforms of CBR3 have shown that CBR3 V244 (G allele) catalyzes the synthesis of cardiotoxic DOXOL at 2.6 times the rate catalyzed by CBR3 M244 [3]. Consistent with these data, we hypothesized that adult breast cancer patients homozygous for the *CBR3* V244M G allele would exhibit worsening cardiac function following DOX treatment when compared with patients homozygous for the A allele.

## Methods

### Study design and patient selection

We performed a prospective single arm observational pharmacogenetic study to examine whether *CBR3* V244M genotype status is associated with changes in echocardiographic measures of cardiomyopathy in breast cancer patients treated with standard dose anthracyclines. We recruited 155 patients with breast cancer receiving treatment with DOX (total cumulative dose: 240 mg/m^2^) at Roswell Park Comprehensive Cancer Center (Buffalo, NY) during the period from 2012 to 2019. To be included in this study, subjects met the following conditions: 1) > 18 years of age, 2) confirmed diagnosis of breast cancer, 3) adequate bone marrow reserve (ANC > 1500/μL, platelet count > 100,000/μL, hemoglobin > 10 g/dL), 4) adequate liver function (serum bilirubin, ALT, AST, and alkaline phosphatase < 1.5x upper limit of normal), and 5) left ventricular ejection fraction (LVEF) within the institutional limits of normal to proceed with DOX administration. Subjects were excluded from the study for the following: 1) uncontrolled intercurrent illness including, but not limited to, ongoing or active infection, symptomatic congestive heart failure, unstable angina pectoris, cardiac arrhythmia, or psychiatric illness/social situations that would limit compliance with study requirements, 2) pregnant or nursing, 3) unwilling or unable to follow protocol requirements, and 4) any condition which in the investigator’s opinion deemed the subject an unsuitable candidate to receive DOX. The protocol was approved by the institutional review boards at Roswell Park and the University at Buffalo. All enrolled patients provided written informed consent. Both men and women and members of all races and ethnic groups were eligible and recruited for this study.

Medical history, physical examination, hematology, chemistry, echocardiogram, and whole blood samples for genetic testing were collected at baseline (defined as within four weeks prior to first dose of DOX). Echocardiographic measurements were performed at baseline and at 6 months of DOX treatment (± 2 weeks) at Buffalo General Medical Center. The clinical variables recorded from each patient’s electronic medical record included age, race, adjuvant trastuzumab treatment and cumulative anthracycline and cyclophosphamide dose. Cardiovascular risk factor covariates such as hypertension, hyperlipidemia, and diabetes were classified as present if both diagnosis and treatment were identified in the medical chart prior to their first echocardiogram. Other cardiovascular risk factors such as smoking history, left-sided radiation and obesity were also recorded. Concurrent use of angiotensin converting enzyme (ACE) inhibitors, angiotensin II receptor blockers (ARBs), beta blockers or statins were included as categorical covariates.

### DNA isolation and quantification

Genomic DNA (gDNA) was isolated from 2 mL of whole blood (deidentified with a unique sample ID) using the QIAamp blood Midi kit (Qiagen, Hilden, Germany) according to the manufacturer’s instructions. gDNA concentration and purity were assessed with a NanoDrop™ One spectrophotometer (Thermo Fisher Scientific, Waltham, MA).

### Genotyping

*CBR3* V244M (rs1056892) was analyzed using predesigned TaqMan genotyping assays (Thermo Fisher Scientific) as per the manufacturer’s guidelines. Genotyping reactions were performed in duplicates and included positive genotyping controls in a Bio-Rad iQ5 thermal cycler (Bio-Rad, Hercules, CA). The absence of contamination was ensured using no gDNA templates (NTC control) for each TaqMan genotyping assay. Genotyping data were collected and analyzed with the IQ5 Software (Bio-Rad).

### Measurements of cardiac function

Of the 155 patients recruited to the study, 92 patients received an echocardiogram at baseline (t_0_) and at 6 months (t_6_) of follow up after DOX treatment at Buffalo General Medical Center. Studies were uploaded and examined offline using the vendor independent TomTec software module (TOMTEC USA, Chicago, IL). Apical two-chamber and four-chamber echocardiographic images were used in TomTec software (TomTec AutoLV, Version; Image-Com5 5.5.4.467461) to automatically calculate volumes and LVEF using Simpson’s biplane rule. The accuracy of the volumetric measurements was improved through manual adjustments of the endocardial borders in end systole and end diastole. Volumetric indices were evaluated by normalizing the cardiac volumes to the body surface area (BSA). Investigators performing the echocardiogram analysis were blinded to all patient information. Cardiotoxicity was defined according to the guidelines from the Cardiac Review and Evaluation Committee of Trastuzumab-associated Cardiotoxicity (CREC), that is, either a reduction of LVEF ≥ 5 to < 55% with symptoms of heart failure or an asymptomatic reduction of LVEF ≥ 10 to < 55% [4].

### Statistical analysis

A two-way ANOVA was performed to test the effect of DOX-treatment and *CBR3* V244M genotype on left ventricular systolic function and their relationship to LVEF itself. Differences in quantitative endpoints (e.g., LVEF, stroke volume index (SVI), end systolic volume index (ESVI), end diastolic volume index (EDVI)) between *CBR3* genotype groups (GG, AG, AA) were assessed using one-way ANOVA and Tukey’s post hoc test. Statistical tests were performed to identify covariates that show significant differences (*p* < 0.05) between genotype groups. Fisher’s exact test was used for categorical variables: trastuzumab treatment, smoking, hypertension (HTN), diabetes, hyperlipidemia, ACE inhibitor use, ARB use, beta blocker use, statin use, and race (dichotomized to non-white [0] and white [1]). One-way ANOVA was used to detect differences between genotype groups for the continuous variables: age, BMI, pre-BSA, anthracycline dose (mg), and cytoxan dose (mg). The cutoff for statistical significance was set at *p* < 0.05. All statistical tests were carried out using SAS Version 9.2 statistical software (Cary, NC).

Multiple linear regression models were developed to model the change in LVEF over 6 months, ∆*LVEF* = *LVEF*_*t*0_ − *LVEF*_*t*6_, was used as the dependent variable [5]. Models were of the form: ∆*LVEF* = *β*_0_ + *β*_1_ ∙ *Geno* + … + *β*_*p*_ ∙ *ARB* + *ε*, where *β*_0_ is an intercept term, and *ε* is an error term. A range of variables were considered as covariates in this model, including age, race, genotype, statins, smoking status, HTN, radiation side, beta blockers, ACE inhibitors and ARB. Other measures were not considered because of low prevalence. Exhaustive subset selection was performed to identify an optimal parsimonious linear model for ∆*LVEF* that is constructed from a reduced set of features [6]. Standard hypothesis tests were performed on the regression coefficients to assess their significance. All models were fit using the R programming language (https://cran.r-project.org/) and predictor effect plots were generated using the “effects” package in R [7].

## Results

### Patients baseline characteristics and demographics

Between 2012 and 2019, a total of 155 patients with a median age of 52 years (range, 24 to 79 years) were recruited to the study and treated with DOX by sequential administration. Of this initial cohort, several patients were excluded from the final analysis due to lack of two complete echocardiograms. In 38 patients who underwent reconstructive surgery between their first and second echocardiogram, the implanted tissue expander overlying the anterior mediastinal space caused impairment of the echocardiographic acoustic window, obscuring a large portion of the left ventricular cavity and endocardial borders in the 6-month study. Twenty additional patients either refused the second study or were noncompliant with their follow-up visit. Two additional patients were removed from the study, one who died prior to the second echocardiogram and one who was found to have metastatic disease necessitating a different treatment regime. Two patients had software processing issues with their echocardiogram data. One patient was not genotyped. After these withdrawals, 92 patients were eligible for echocardiographic assessment of cardiotoxicity following DOX treatment (Table 1). Most of the patients had a good Eastern Cooperative Oncology Group performance score (66% ECOG PS of 0, 34% ECOG PS of 1). At the start of treatment, all 92 patients had normal systolic function (LVEF > 55%).

**Table 1:** Patient demographics

	***CBR3*** Genotype				
	AA	AG	GG	All Patients	***p*** Valve
	(*N* = 13)	(*N* = 53)	(*N* = 26)	(*N* = 92)	
	n (%)	n (%)	n (%)	n (%)	
**Chemotherapy, mean ± SD**					
Anthracycline dose, mg	461 ± 77	424 ± 60	439 ± 49	433 ± 61	0.168
Cytoxan dose, mg	4645 ± 827	4315 ± 579	4397 ± 517	4384 ± 615	0.979
Treatment days	4 ± 0	3.9 ± 0.37	4.0 ± 0.19	3.9 ± 0.4	0.2849
**Trastuzumab (adjuvant)**	1 (8%)	4 (8%)	3 (11%)	8 (9%)	0.859
**Chest radiation, left-side**	6 (46%)	28 (53%)	12 (46%)	46 (50%)	0.732
**Sex, female**	11 (85%)	53 (100%)	26 (100%)	90 (98%)	0.002*
**Age, years**					
Mean ± SD	55 ± 12	51 ± 12	51 ± 12	52 ± 11	0.692
Range	33–76	24–73	32–73	24–76	
**Race**					
Caucasian	10 (77%)	48 (91%)	23 (89%)	81 (88%)	0.396
African American	1 (8%)	4 (8%)	3 (11%)	8 (9%)	
Hispanic	0	0	0	0	
Asian	1 (8%)	0	0	1 (1%)	
Mixed	1 (8%)	1 (2%)	0	2 (2%)	
**Comorbidities**					
Hypertension	5 (38%)	15 (28%)	5 (19%)	25 (27%)	0.386
Diabetes	1 (8%)	3 (6%)	2 (7%)	6 (6%)	0.938
Hyperlipidemia	6 (46%)	9 (17%)	5 (19%)	21 (23%)	0.069
Smoking history	4 (31%)	21 (40%)	10 (37%)	35 (38%)	0.838
**Medication**					
ACE inhibitor	1 (8%)	5 (9%)	3 (11%)	9 (10%)	0.126
ARB	2 (15%)	3 (6%)	2 (7%)	7 (8%)	0.492
Beta blocker	2 (15%)	4 (8%)	1 (4%)	7 (8%)	0.423
Statin	4 (31%)	6 (11%)	0 (0%)	10 (11%)	0.014*

Differences between groups were compared using Fisher’s exact for categorical variables and one-way ANOVA for continuous variables

*p < 0.05, indicates significant differences between subgroups

*Abbreviations*: *SD* standard deviation, *ACE* angiotensin-converting enzyme, *ARB* angiotensin II receptor blocker

Cardiovascular assessment prior the start of treatment revealed no history of coronary artery disease (CAD), myocardial infarction, heart failure and uncontrolled hypertension in any of the 92 study patients. 2% of patients had a pre-existing history of rhythm disturbances (atrial flutter and supraventricular tachycardia). Assessment of cardiovascular risk factors revealed that the following were most common: smoking (38%), hypertension (HTN, 27%), hyperlipidemia (defined as fasting cholesterol > the upper normal limit, 23%), and type II diabetes mellitus (7%). A percentage of patients were also on the following medications: ACE inhibitors (10%), ARBs (8%), beta blockers (8%) and statins (10%). In addition to standard adjuvant chemotherapy with an anthracycline, cytoxan and taxane-based regimen, 92% of patients received radiation as part of their treatment (49% left sided; 44% right sided), and 8% received treatment with trastuzumab (Table 1).

### Association of DOX-induced cardiotoxicity with *CBR3* V244M genotype status

**Fig. 1:**
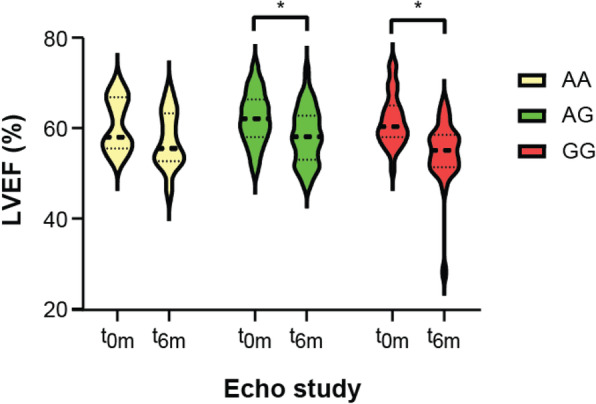
Left ventricular systolic function in adult breast cancer patients treated with DOX by echocardiogram at baseline and 6 months. We obtained apical two-chamber and four-chamber echocardiographic images at baseline (prior to DOX treatment; t_0m_) and 6 months (±2 weeks) following the initiation of DOX therapy (t_6m_). These images were used to calculate LVEF using Simpson’s biplane rule by investigators blinded to all patient data. The data was graphed as a violin plot showing the median (thick dashed line), quartiles (dotted line) and overall distribution of LVEF by *CBR3* V244M genotype (AA, AG, GG) at t_0m_ and t_6m_. *p* < 0.0001, two-way ANOVA

**Fig. 2:**
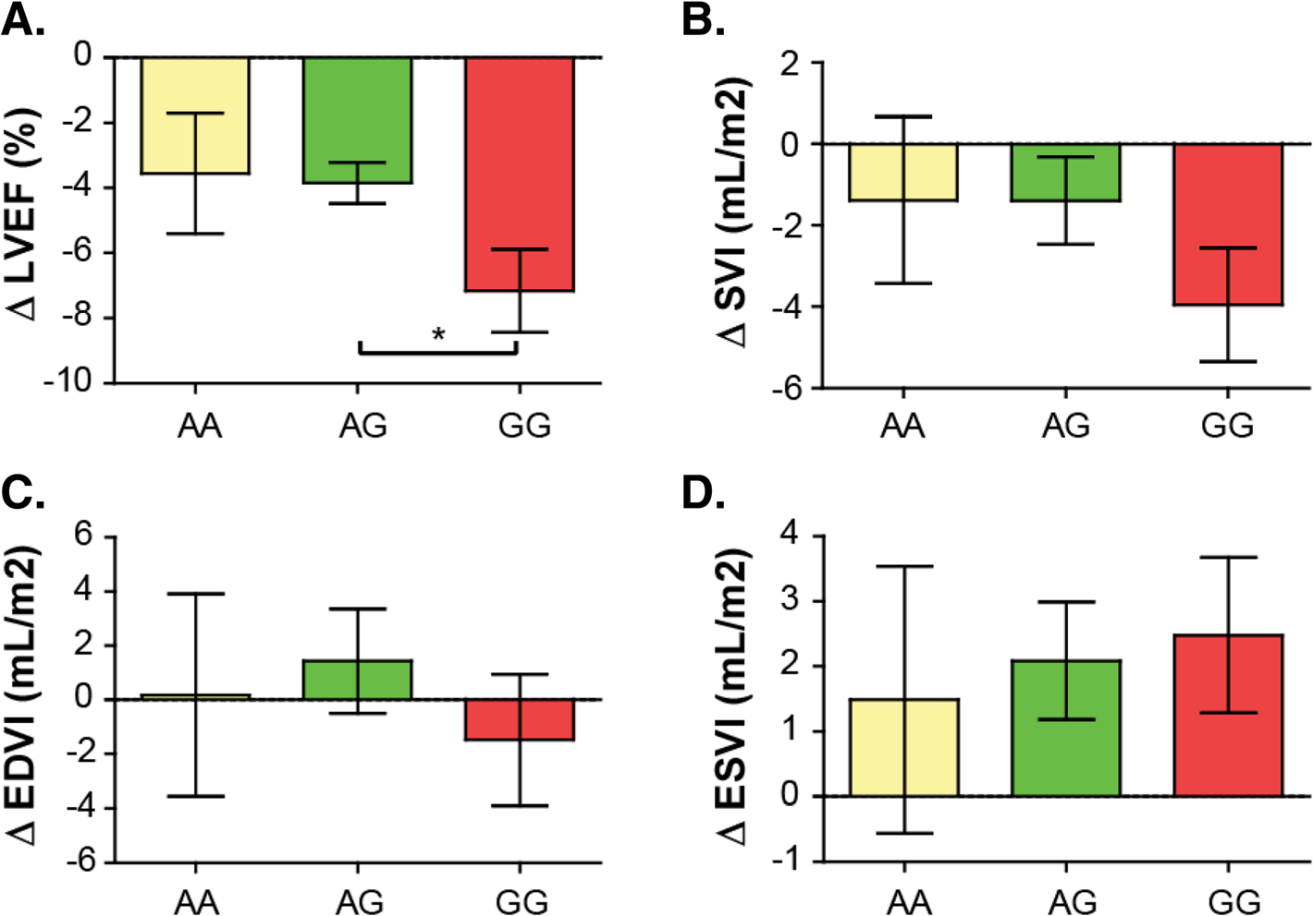
Comparison of ΔLVEF, SVI, EDVI and ESVI between *CBR3* V244M genotypes. Baseline and 6-month echocardiographic images were used to calculate volumes and LVEF by blinded investigators. Volumetric indices were evaluated by normalizing cardiac volumes to body surface area (BSA). Baseline metrics were subtracted from 6-month values to calculate changes in **a**) LVEF, **b**) stroke volume index (SVI), **c**) end systolic volume index (ESVI) or **d**) end diastolic volume index (EDVI) between *CBR3* V244M genotypes (AA, AG, GG) after DOX treatment. *p* = 0.040, one-way ANOVA. Data expressed as mean ± SEM

We compared left ventricular systolic function from pre- and post-DOX treatment echocardiograms in each *CBR3* V244M genotype group and found a highly significant effect of DOX treatment on left ventricular systolic function (F[1,89] = 50.33, *p* < 0.0001, two-way ANOVA). Breast cancer patients with *CBR3* GG and AG genotypes experienced a statistically significant reduction in LVEF at 6 months (t_6m_) following initiation of DOX treatment compared with their pre-DOX baseline study (t_0m_) (*p* < 0.001) (Fig. 1). While AA trended towards a decrease in LVEF following DOX-treatment at the 6-month echocardiogram, it was not statistically significant (*p* = 0.072). *CBR3* V244M genotype status did not show a significant effect on ejection fraction (EF) alone (F[2,89] = 1.369, *p* = 0.260, two-way ANOVA) but had a significant effect on the progressive loss of left ventricular systolic function in patients undergoing DOX therapy at 6-months (interaction, F[2,89] = 3.410, *p* = 0.037, two-way ANOVA). Patients homozygous for the *CBR3* V244M G allele (CBR3 V244) exhibited a further statistically significant decrease in LVEF at 6 months following DOX therapy, suggestive of cardiotoxicity, in comparison with patients with the AG genotype. Pair-wise Tukey testing revealed that patients with the *CBR3* GG genotype had a significantly lower EF compared to those with the AG genotype (3.32% EF difference, 95% CI [− 6.52, − 0.12], *p* = 0.0397, one-way ANOVA), and trended toward a worsening EF compared with AA patients (3.61, 95% CI [− 8.15, 0.924], *p* = 0.145, one-way ANOVA) (Fig. 2a). There was a trend in decreased stroke volume index (SVI) for GG vs. AG (− 2.35 ml/m^2^, 95% CI [− 6.51, 1.82], *p* = 0.375, one-way ANOVA) and GG vs. AA (− 2.66 ml/m^2^, 95% CI [− 8.44, 3.11], *p* = 0.518, one-way ANOVA) which did not reach statistical significance (Fig. 2b). There was no significant difference between ESVI (1-way ANOVA, F(2,84) = 0.085, *p* = 0.918, one-way ANOVA) (Fig. 2c) or end diastolic volume index (EDVI, 1-way ANOVA, F(2,84) = 0.213, *p* = 0.808, one-way ANOVA) (Fig. 2d) between genotype groups (Fig. 1). While there was a significant association between genotype and declining LVEF, we did not identify a significant association with cardiotoxicity as measured by *CBR3* V244M genotype status and risk for anthracycline-related cardiotoxicity (by definition, an asymptomatic reduction of LVEF ≥ 10 to < 55%) (odds ratio (OR) = 2.55, 95% CI [0.26, 25.17], *p* = 0.424 for GG vs. AA; OR = 1.43, 95% CI [0.15 to 13.26], *p* = 0.754 for GA vs. AA).

We found no statistically significant difference in age, history of cardiovascular diseases, risk factors for cardiovascular disease, cumulative DOX dose, or concurrent use of beta blockers or ACE inhibitors between *CBR3* genotype groups (Table 1). However, higher usage of statins was detected in patients with *CBR3* AA genotype compared to patients with AG or GG genotypes (31% vs. 11 and 0%, respectively, *p* = 0.014, Fisher’s exact test), despite no statistically significant difference in the incidence of hyperlipidemia between AA, AG, and GG genotype groups (46, 17, and 19%, respectively, *p* = 0.069, Fisher’s exact test). Patients on chronic statins were more often older (62 ± 8 vs. 50 ± 11, mean ± SD, *p* < 0.001, unpaired t test), taking beta blockers (30% vs. 5%, *p* = 0.033, Fisher’s exact test), taking ARBs (30% vs. 5%, p = 0.033, Fisher’s exact test), and carrying a diagnosis of hyperlipidemia (100% vs. 12%, *p* < 0.001, Fisher’s exact test) compared with patients not using statins (Supplementary Table 1). In the subset of patients receiving statins, there was no statistically significant change in LVEF at baseline and six months after initiating anthracycline therapy (60% ± 4.7 vs. 57% ± 5.6, *p* = 0.25, unpaired t test). In those patients not receiving statins however, there was a statistically significant decrease in LVEF from 62% ± 5.8 at baseline to 57% ± 6.7 over a similar 6-month interval (*p* < 0.001, unpaired t test). Significance was not observed in the multiple regression model, which is likely due to the small sample size.

**Fig. 3:**
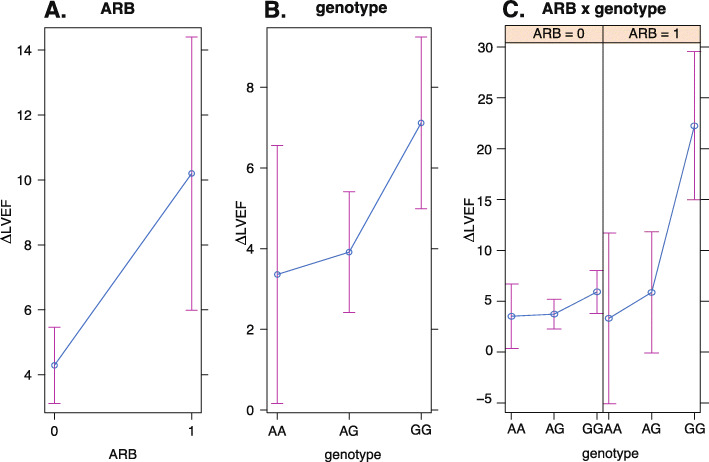
Regression effect plots for the significant regression variables in the ΔLVEF model. The regression effect plot for ΔLVEF is shown for **a**) ARB (*p* = 0.019) and **b**) *CBR3* V244M genotype (GG, *p* = 0.029), and **c**) the interaction between ARB use and *CBR3* genotype

Exhaustive subset selection resulted in an optimal for multiple linear regression model (*p* = 0.0098, adjusted R-squared = 0.1429) for ΔLVEF that included the dependent variables: age, ARB, and *CBR3* genotype. Hypothesis tests on the regression coefficients revealed significance for ARB usage (*p* = 0.0187) and genotype (*p* = 0.0290) (Fig. 3a,b). Estimated regression coefficients and *p*-values for this model are shown in Table 2. Predictor effect plots [7] from the regression model show that there is a clear increase in ΔLVEF in the ARB group after accounting for other covariates in the model (Fig. 3a). Moreover, there is a clear difference in ΔLVEF by *CBR3* genotype, with the homozygous GG group exhibiting a major increase in ΔLVEF (Fig. 3b). Although the ARB group is small (*n* = 7) there is a clear increase in ΔLVEF in the homozygous GG group (Fig. 3c). In fact, the two patients in the GG group exhibited relatively large ΔLVEF (ΔLVEF = 12.6 and ΔLVEF = 31.9).

**Table 2:** Summary statistics for the regression model for ΔLVEF

Coefficients:				
	Estimate	Std. Error	T value	Pr(>ItI)
(Intercept)	1.10562	3.19827	0.346	0.7304
ARB	5.35886	2.23582	2.397	0.0187*
Age	0.02953	0.05256	0.562	0.5757
genoAG	0.91455	1.70261	0.537	0.5925
genoGG	4.13718	1.86285	2.221	0.0290*

Significance codes: 0 ‘***’, 0.001 ‘**’, 0.01 ‘*’

## Discussion

We performed a prospective pharmacogenetic study to analyze the impact of the functional *CBR3* V244M polymorphism on the development of acute anthracycline cardiotoxicity in a cohort of 92 breast cancer patients receiving DOX. Importantly, we found a significant association between *CBR3* V244M genotype status and LVEF decline following DOX chemotherapy (central illustration). Patients homozygous for the *CBR3* V244M G allele exhibited a lower LVEF following treatment with DOX when compared with patients with heterozygous AG genotype. These results highlight the *CBR3* V244M G allele (CBR3 V244) as a potential genetic risk factor for the development of anthracycline-induced systolic dysfunction. Importantly, even asymptomatic LV dysfunction carries significant risk of subsequent heart failure and death [8]. As long-term survivorship increases, the incidences of cardiac morbidity and mortality can be expected to increase in DOX recipients as they age [9–11]. While the data did not meet statistical significance for an association with cardiotoxicity at 6 months following DOX therapy, the associated anthracycline-induced decline in LVEF among otherwise young and healthy patients with the GG genotype may serve as a “first hit” that predisposes them to a heightened risk of developing later systolic dysfunction. We know that once damaged, cardiomyocytes have a limited capacity for repair. In a multiple hit paradigm, patients with the *CBR3* GG genotype previously treated with DOX may develop earlier and more severe cardiomyopathy than patients with the *CBR3* AA or AG genotypes after undergoing any additional cardiac insult (i.e., exposure to other cytotoxic agents or development of ischemic heart disease). Additional prospective longitudinal studies which follow these variables over the course of years to several decades would be helpful in delineating this hypothesis.

A number of prior studies have looked at the usefulness of several cardiovascular drugs for the primary prevention of chemotherapy-induced cardiotoxicity [12]. Current data suggest that beta blockers (carvedilol and nebivolol) [13–15], ACE inhibitors (enalapril) [16], ARBs (candesartan) [17], as well as combinations of these drugs (carvedilol and enalapril) [18] may prevent left ventricular dysfunction. While we documented no significant difference between beta blocker or ACE use between *CBR3* genotype groups, we did observe a higher incidence of statin use amongst patients with the *CBR3* AA genotype. Chronic statin administration has been suggested to attenuate early anthracycline-associated declines in left ventricular ejection fraction [19, 20], likely via pleiotropic effects such as the reduction of oxidative stress, inflammatory cytokines and circulating neurohormones [21, 22]. In our study, individuals receiving statins were older, more often had hyperlipidemia, and more frequently took beta blockers and ARBs than the subset of patients not taking statins. Interestingly however, we saw no difference in statin use between GG and AG genotypes, the comparison of which had the most significant difference in LVEF following DOX-therapy.

Multiple regression models allowed us to assess the simultaneous effect of multiple predictors on ΔLVEF. Our models show that ΔLVEF is best explained by a combination of age, ARB usage and *CBR3* genotype. Regression models were suggestive of a significant interaction between *CBR3* genotype and ARB, but a larger sample size would be needed to explore this further.

Genetic screening for high-risk variants may help identify a subpopulation of cancer patients at increased risk for chemotherapy induced cardiomyopathy who may be eligible for initiation of preventative cardioprotective medications (ACEi, beta blockers), alternations in chemotherapy dose regimes, and closer surveillance with cardiac biomarkers during and after treatment with anthracycline or trastuzumab. It may also enable identification of novel biomarkers for risk stratification and surveillance based upon the specific polymorphism.

While the concept of starting all cancer patients on cardioprotective strategies prior to cancer treatment is gaining interest given the encouraging results of such trials as OVERCOME and ICOS-ONE, there are concerns of subjecting low-risk patients to unnecessary adverse events such as hypotension and renal dysfunction [18, 23]. Additionally, hemodynamic compromise may also occur as cancer patients are susceptible to sepsis and dehydration [24]. Having the ability to target cardioprotective strategies to a subpopulation of patients at higher risk for development of cardiotoxicity, potentially identified by genetic screening and/or circulating biomarkers, would help to mitigate the risk of adverse events.

Our current findings in adult patients undergoing DOX treatment for breast cancer are in line with our prior findings in survivors of pediatric cancers. In a nested case-control pilot study of patients enrolled in the Childhood Cancer Survivor Study, we identified a positive trend between *CBR3* V244M genotype status and risk for anthracycline-related heart failure (odds ratio (OR) = 8.16 and *p* = 0.056 for GG vs. AA; OR = 5.44 and *p* = 0.092 for GA vs. AA) [25]. In a later study looking at a larger cohort of survivors with and without cardiomyopathy, we found that even at low doses of anthracycline (1–250 mg/m^2^) patients with *CBR3* GG genotypes had a higher risk of cardiomyopathy when compared with individuals with *CBR3* GA/AA genotypes unexposed to anthracyclines (OR = 5.5, *p* = 0.003), or exposed to < 250 mg/m^2^ (OR = 3.3, *p* = 0.006) [3].

Since our initial studies, there have been several pharmacogenetic analyses focused on the *CBR3* V244M variant in adult cohorts treated with anthracyclines. While a number of studies have identified the *CBR3* V244M variant as a genetic risk factor for anthracycline cardiotoxicity [26] others have detected no associations [27, 28]. Interestingly, a cross-sectional observational study of anthracycline-induced cardiomyopathy in a cohort of women with breast cancer found the *CBR3* V244M AA genotype more often in patients with systolic dysfunction than in cases with normal LVEF > 55% [29]. The study showed a significantly increased risk of cardiomyopathy with the AA genotype in either an additive (OR = 2.50; 95% CI: 1.22–5.11; *p* = 0.012) or recessive (OR = 6.19; 95% CI: 1.94–19.76; *p* = 0.002) genetic model [29]. These findings are in contrast with those from the current study and our prior work on survivors of pediatric cancers. Our findings are consistent with evidence from kinetic studies - i.e., CBR3 V244 (G allele) has higher catalytic activity for anthracycline substrates than CBR3 M244 (A allele) - and a pharmacokinetic model of myocardium exposure which suggest that patients with *CBR3* GG genotype treated with DOX have a higher simulated risk of heart failure [30, 31].

Differences in patient age, tumor type and treatment regimens may account for the disparate results between our current prospective study and the observational study conducted by Hertz et al. For example, 20% of the patients in their cohort were treated with sequential use of cardiotoxic trastuzumab and approximately 40% of patients received left sided chest radiation. In our study, while 50% of patients had left sided chest radiation, only 8% of patients received adjuvant trastuzumab. These differences may contribute to the inability to replicate these associations between cohorts as genetic predictors of anthracycline-induced cardiotoxicity may be distinct among tumor types and treatment regimes. Going forward, additional large prospective clinical trials with cohorts of similar backgrounds and treatments followed for longer periods of time will need to be conducted to further elucidate the importance of *CBR3* V244M as a predictor for cardiotoxicity.

### Study strengths and limitations

We were forced to remove 25% (38/155) of study participants from the final analysis due to poor acoustic windows in the 6-month follow-up echocardiogram secondary to recent placement of tissue expanders. Having a larger sample size would be helpful in exploring interactions between variables and understanding the differences between statin and non-statin users. In addition, while any subject loss is undesirable as it leads to loss of statistical power, it can also lead to selection bias if the probability of being removed from the study or lost to follow-up depends on the outcome to be measured. However, as none of our patients exhibited symptoms of heart failure, it is unlikely that those who chose to undergo reconstructive surgery were any less (or more) likely to have asymptomatic left ventricular systolic dysfunction.

It is important to note that a decrease in LVEF is a late manifestation of cancer therapy-associated cardiotoxicity. Echocardiographic imaging without myocardial deformation indices would miss early detection of cardiotoxicity before it manifests as a reduction in LVEF or heart failure symptoms. Future studies incorporating strain would be more sensitive for the detection of subclinical LV systolic dysfunction. Additionally, inclusion of surveillance biomarkers such as troponin I (TnI), B-type natriuretic peptide (BNP), and myeloperoxidase may augment identification of subpopulations of patients at high risk of cardiotoxicity.

An additional limitation of the study centers on the study design. Prospective cohort studies are inherently less useful for conditions with long latencies. If the asymptomatic reduction in LVEF amongst *CBR3* GG patients undergoing DOX treatment predisposes them to more significant cardiotoxicity in the future following “additional hits”, the 6-month follow-up period will miss the longer latency for associated cardiotoxicity.

Despite these limitations, this study has several strengths. The design of our prospective cohort study reduced the issue of selection bias and provided clarity on the temporal sequence between DOX-exposure and left ventricular systolic function. We implemented standardized treatment regimens with minimum confounding from trastuzumab. Our cohort was comprised of a relatively homogenous population of relatively young women (mean age of 52 years) with few comorbidities. Changes in systolic function in this population are especially important given the increased survivorship and potential for additional cardiovascular insults later in life to which they may be more prone to developing a cardiomyopathy. Finally, all echocardiograms were performed and analyzed by the same investigator, eliminating interobserver bias, and were conducted blinded to clinical and genomic data.

## Conclusions

In our study of 92 breast cancer patients treated with DOX, the *CBR3* V244M variant was found to be a significant independent predictor of LV systolic function. Patients with *CBR3* GG and AG genotypes exhibited statistically significant declines in LVEF at 6 months following DOX treatment. The *CBR3* GG genotype was associated with a further reduction in LV systolic function when compared with the heterozygous AG genotype, highlighting G as the *CBR3* risk allele.

### Perspectives

*Competency in Medical Knowledge:* As cancer outcomes have improved, the potential cardiotoxicity of chemotherapeutic agents has become an important issue for survivorship. In addition to screening and treating patients for modifiable risk factors associated with LV systolic dysfunction, identification of genetic polymorphisms that convey risk of chemotherapy-induced cardiotoxicity are needed to design risk-based strategies for surveillance and facilitate informed therapeutic decision-making regarding prevention and treatment.

*Translational Outlook:* Anthracyclines, such as DOX, are a mainstay in chemotherapy but are associated with cardiovascular side-effects. We previously demonstrated an association of the *CBR3* V244M polymorphism with a late manifestation of cardiac dysfunction in long term survivors of pediatric cancers [3]. Additional genetic analyses are needed in large prospective cohorts of adult cancer patients treated with anthracyclines and followed for extended periods of time to further investigate the role of *CBR3* V244M across patients of different ages, cancer types, and treatment regimes. Future clinical trials would also benefit from screening biomarkers (troponin I, BNP) prior to chemotherapy initiation and during treatment, as well as inclusion of strain imaging to enable identification of subclinical cardiac dysfunction prior to a reduction in LVEF. In addition, there is a need for more comprehensive algorithms for risk prediction that incorporate both clinical and genetic data [32].

## Supplementary Information

**Additional file 1: Supplementary Table 1**. Sub analysis of statin users.

## Data Availability

The datasets used and/or analyzed during the current study are available from the corresponding authors on reasonable request.

## Abbreviations

ACE: Angiotensin converting enzyme
AKR: Aldo-keto reductase
ALT: Alanine aminotransferase
ANC: Absolute neutrophil count
ARB: Angiotensin receptor blocker
AST: Aspartate aminotransferase
BMI: Body mass index
BNP: B-type natriuretic peptide
BSA: Body surface area
CAD: Coronary artery disease
CBR3: Carbonyl reductase 3
CI: Confidence interval
DOX: Doxorubicin
ECOG PS: Eastern Cooperative Oncology Group Performance Status Scale
EDVI: End diastolic volume index
ESVI: End systolic volume index
EF: Ejection fraction
gDNA: Genomic DNA
HER2: Human epidermal growth factor receptor 2
LVEF: Left ventricular ejection fraction
NTC: No template control
OR: Odds ratio
ROS: Reactive oxygen species
SNP: Single nucleotide polymorphism
SVI: Stroke volume index
TnI: Troponin I
