# Supplementary material for: CBR3 V244M is associated with LVEF reduction in breast cancer patients treated with doxorubicin
Source: Cardiooncology. 2021 May 11;7:17. doi: 10.1186/s40959-021-00103-0 (PMC8111996; doi:10.1186/s40959-021-00103-0)
Supplement: Supplementary file 1 — Additional file 1: Supplementary Table 1. Sub analysis of statin users. [file 40959_2021_103_MOESM1_ESM.docx]

**Supplementary Table I. Sub analysis of statin users**

|  | **Statin users** | **Non-statin users** | ***p* Valve** |
| --- | --- | --- | --- |
|  | (N=10) | (N=82) |  |
|  | n (%) | n (%) |  |
| **Chemotherapy, mean ± SD** |  |  |  |
| Anthracycline dose, mg | 433 ± 62 | 433 ± 61 | 1.00 |
| Cytoxan dose, mg | 4473 ± 628 | 4373 ± 612 | 0.612 |
| Treatment days | 3.9 ± 0.29 | 4.0 ± 0.31 | 0.314 |
| **Trastuzumab, adjuvant** | 1 (10%) | 7 (9%) | 1.00 |
| **Chest radiation, left-side** | 5 (50%) | 41 (50%) | 1.00 |
| **Sex, female** | 9 (90%) | 81 (99%) | 0.221 |
| **Age, years** |  |  |  |
| Mean ± SD | 62 ± 8 | 50 ± 11 | 0.001 |
| Range | 48-76 | 24-73 |  |
| **Race** |  |  |  |
| Caucasian | 10 (100%) | 61 (74%) | 0.345 |
| African American | 0 | 8 (10%) |  |
| Hispanic | 0 | 0 |  |
| Asian | 0 | 1 (1%) |  |
| Mixed | 0 | 2 (2%) |  |
| **Comorbidities** |  |  |  |
| Hypertension | 5 (50%) | 20 (24%) | 0.154 |
| Diabetes | 1 (10%) | 5 (6%) | 0.536 |
| Hyperlipidemia | 10 (100%) | 10 (12%) | 0.001* |
| Smoking history | 3 (30%) | 32 (39%) | 0.529 |
| **Medication** |  |  |  |
| ACE inhibitor | 1 (10%) | 8 (10%) | 1.00 |
| ARB | 3 (30%) | 4 (5%) | 0.033* |
| Beta blocker | 3 (30%) | 4 (5%) | 0.033* |
| **CBR3 V244M Genotype** |  |  |  |
| AA | 4 (40%) | 9 (11%) | 0.043* |
| AG | 6 (60%) | 47 (57%) | 1.00 |
| GG | 0 (0) | 26 (31%) | 0.057 |

Differences between groups were compared using Fisher’s exact for categorical variables and unpaired two-tailed t test for continuous variables.

*p<0.05, indicates significant differences between groups
